# A Qualitative Signature to Identify *TERT* Promoter Mutant High-Risk Tumors in Low-Grade Gliomas

**DOI:** 10.3389/fmolb.2022.806727

**Published:** 2022-04-14

**Authors:** Weicheng Zheng, Ruolan Zhang, Ziru Huang, Jianpeng Li, Haonan Wu, Yuwei Zhou, Jinwei Zhu, Xianlong Wang

**Affiliations:** ^1^ College of Bioinformatics Science and Technology, Harbin Medical University, Harbin, China; ^2^ Department of Bioinformatics, School of Medical Technology and Engineering, Key Laboratory of Medical Bioinformatics, Key Laboratory of Ministry of Education for Gastrointestinal Cancer, Fujian Medical University, Fuzhou, China

**Keywords:** glioma, TERT promoter, biomarker, transcriptome, relative expression orderings

## Abstract

**Background:** Telomerase reverse transcriptase promoter (*TERT*-p) mutation has been frequently found, but associated with contrary prognosis, in both low-grade gliomas and glioblastomas. For the low-grade gliomas (Grades II-III), *TERT*-p mutant patients have a better prognosis than the wildtype patients, whereas for the GBMs (Grade IV), *TERT*-p mutation is related to a poor prognosis. We hypothesize that there exist high-risk patients in LGGs who share GBM-like molecular features, including *TERT*-p mutation, and need more intensive treatment than other LGGs. A molecular signature is needed to identify these high-risk patients for an accurate and timely treatment.

**Methods:** Using the within-sample relative expression orderings of gene pairs, we identified the gene pairs with significantly stable REOs, respectively, in both the *TERT*-p mutant LGGs and GBMs but with opposite directions in the two groups. These reversely stable gene pairs were used as the molecular signature to stratify the LGGs into high-risk and low-risk groups.

**Results:** A signature consisting of 21 gene pairs was developed, which can classify LGGs into two groups with significantly different overall survival. The high-risk group has a similar genetic mutation profile and a similar survival profile as GBMs, and these high-risk tumors may progress to a more malignant state.

**Conclusion:** The 21 gene-pair signature based on REOs is capable of identifying high-risk patients in LGGs and guiding the clinical choice for appropriate and timely intervention.

## Introduction

Primary tumors account for more than half of central nervous system tumors, of which gliomas are the most frequent, accounting for 45–50% of all primary malignant brain tumors (Williams et al., 2018; Ostrom et al., 2019). Based on histopathological criteria, gliomas are classified into low-grade gliomas (LGGs) and glioblastoma (GBMs, Grade IV). The LGGs consist of oligodendroglioma (OD, Grades II-III) and astrocytoma (A, Grades II-III) (Louis et al., 2016). A frustrating reality is that not much progress has been made in prediction and treatment of gliomas for a long time (Filbin and Suva, 2016). Gliomas have a characteristic diffusely infiltrative pattern of growth in brain, and it is impossible to achieve complete resection (Cancer Genome Atlas Research et al., 2015). Prediction of the clinical outcome is often inaccurate because current standards are often subjective and dependent on pathologists’ experience, and it is almost impossible to distinguish the mixed histological appearance of glioma tissues (Cancer Genome Atlas Research et al., 2015). High interobserver variability results in high risk of misclassification, which has documented serious clinical consequences and may affect the choice of treatment options and prognosis of patients (Foote et al., 2015).

In recent years, various molecular markers have been discovered for gliomas, including isocitrate dehydrogenase 1 and 2 (*IDH 1*/*2*) mutations (Lu et al., 2012), codeletion of chromosome arms 1p and 19q (1p19q co-del) (Cairncross et al., 2013; van den Bent et al., 2013; Buckner et al., 2017) and telomerase reverse transcriptase promoter (*TERT*-p) hotspot mutations (Chan et al., 2015; Foote et al., 2015; Heidenreich et al., 2015; Masui et al., 2018). The 2016 World Health Organization (WHO) Classification of Tumors of the Central Nervous System (CNS) integrated *IDH* and 1p/19q co-del into clinical diagnosis (Louis et al., 2016). These molecular diagnostic markers are challenging our prior assumptions concerning the definitions of gliomas and becoming one of the most important factors for glioma prognosis (Buckner et al., 2017).

TERT is an enzyme that maintains the length of telomeres (O'Sullivan and Karlseder, 2010; Heidenreich and Kumar, 2017). Approximately 83% of primary GBMs (Killela et al., 2013) and 79.3% of OD (Killela et al., 2014) harbor a mutation of C to T in a mutually exclusive manner in the promoter of *TERT* at either -124 or -146 bp upstream from the transcription start site (Heidenreich et al., 2015). The mutations result in an additional ETS (E26 transformation-specific family transcription factor) binding site recognized by GABPA (GA-binding protein A), which facilitates reactivation of telomerases (Bollam et al., 2018). Previous research found that mutations of *TERT*-p lead to an increase in transcriptional activity by 2–4 times (Heidenreich et al., 2015).

The *TERT*-p mutations mainly occur in tumors from tissues with low rates of self-renewal (Heidenreich and Kumar, 2017). While being first discovered in melanoma, the *TERT*-p mutations have been found defining subsets of patients with adverse disease outcomes in hepatocellular carcinoma, urothelial carcinoma and other tumors (Heidenreich and Kumar, 2017; Heidenreich and Kumar, 2017). The prognostic impact of *TERT*-p mutation in diffuse gliomas appears to be ambivalent (Arita et al., 2016). In LGGs, *TERT*-p mutation is a hallmark for better prognosis, whereas in GBM it tends to be associated with poor prognosis (Reitman et al., 2013; Simon et al., 2015). Therefore, the consequence of *TERT*-p mutation and its interaction with other molecular markers in glioma pathogenesis remain to be fully understood (Bollam et al., 2018). High-throughput genomic sequencing is often used for the detection of *TERT*-p mutations, but the sensitivity is affected by the proportion of tumor cells and sequencing depth.

Treatment of glioma patients is critical to the prognosis. Gliomas of different grades or specific molecular states should receive corresponding treatments. For example, LGGs especially OD are known to be sensitive to procarbazine, lomustine and vincristine (PCV)-based chemotherapy. The response rate of LGG to PCV as an initial therapy ranges from 52 to 100% (Buckner et al., 2017). GBMs are generally treated with temozolomide (TMZ) after resection (Hegi et al., 2005). While *TERT*-p mutations mainly occur in OD and GBM, accurate classification to distinguish them is critical for choosing the right treatment. Misclassified patients are at risk of either over-treatment or under-treatment, which is detrimental to clinical decision-making and patient survival. Under-treatment is one of the most serious problems in cancer treatment and directly related to the survival. There is already ample evidence that some gliomas are misclassified as LGGs. They have malignant molecular alterations, but not yet exhibit histopathological characteristics (Bruner et al., 1997; Olar and Sulman, 2015). These gliomas usually have a strong tendency to develop into more malignant states, but indistinguishable due to their mixed histological appearance. Conventional histopathological diagnosis may suffer from morphological ambiguity and interobserver discordance (Arita et al., 2016). It is necessary to develop a robust and objective molecular marker to identify them.

In this study, we aim to develop a robust molecular marker by comparing the transcriptional profiles of LGGs and GBMs, in order to identify patients with high-grade characteristics (GBM-like patients) from LGGs. For these patients, a more aggressive treatment is recommended.

## Materials and Methods

### Data Sources and Data Preprocessing

The gene expression profiles used in this study were downloaded from various databases. Datasets GSE61374, GSE16011, GSE43388 and GSE68848 were downloaded from the Gene Expression Omnibus (Barrett et al., 2007) (GEO, http://www.ncbi.nlm.nih.gov/geo/). Datasets TCGA-LGG and TCGA-GBM were downloaded from The Cancer Genome Atlas (TCGA, https://cancergenome.nih.gov/) (Hoadley et al., 2018). Dataset E-MTAB-3892 was downloaded from ArrayExpress (http://www.ebi.ac.uk/arrayexpress/). Dataset CGGA was downloaded from the Chinese Glioma Genome Atlas (CGGA, http://www.cgga.org.cn/) (Bao et al., 2014; Zhao et al., 2017; Hu et al., 2018). The GBM-cohort include 301 sample with *TERT* mutant status (Arita et al., 2016). Clinical information for each dataset is shown in Table 1 and Supplementary Table S1. The TCGA dataset was used as the training set, which include 130 grades II and III samples and 23 grade IV samples. The *TERT*-p mutation status of grade IV samples is unknown. In order to further improve the transferability of the classification signature on different profiling platforms, two GEO datasets, GSE16011 (*n* = 159) and GSE61374 (*n* = 133, low-grade samples only) were also used for training.

**TABLE 1:** Clinical characteristics of the training datasets.

	TCGA			GSE16011-61374	
	*TERT*-p mut (*n* = 149)		*TERT*-p wt (*n* = 158)	*TERT*-p mut (*n* = 204)	*TERT*-p wt (*n* = 88)
Grade and Histological Type — no.(%)					
Grade II					
Oligodendroglioma	43 (72.9)	26 (31.7)		3 (21.4)	0 (0.0)
Oligoastrocyma	13 (22)	29 (35.4)		6 (42.9)	7 (15.6)
Astrocytoma	3 (5.1)	27 (32.9)		5 (35.7)	38 (84.4)
Grade III					
Oligodendroglioma	38 (53.5)	9 (12.5)		2 (6.5)	1 (2.3)
Oligoastrocyma	11 (15.5)	20 (27.8)		18 (58.0)	12 (27.9)
Astrocytoma	22 (31)	43 (59.7)		11 (35.5)	30 (69.8)
Grade IV					
Glioblastoma	19 (-)	4 (-)		159 (-)	0 (-)
Age at diagnosis — years					
Mean	50.14 ± 12.6	37.3 ± 12.0		53.0 ± 13.6	39.9 ± 12.4
Range	20–76	14–70		15–81	21–80
Male sex — no./total (%)	79/149 (53.0)	93/158 (58.9)		134/204 (65.7)	56/88 (63.6)
*IDH* status — no. (%)					
*IDH1/2*-mut	93 (62.4)	129 (98.0)		68 (33.3)	77 (87.5)
*IDH*-wt	55 (36.9)	19 (12.0)		105 (51.5)	1 (12.5)
1p19q status — no. (%)					
Co-del	86 (57.7)	2 (1.3)		-	-
No co-del	63 (42.3)	156 (98.7)		-	-
Median survival — months	13.4	17.3		12.7	49.0

### Selection of Differentially Expressed Genes

Differentially expressed genes (DEGs) were selected between the *TERT*-p mutant low-grade gliomas (LGGs) and *TERT*-p mutant GBM (high-grade gliomas) samples using the following procedure. Genes in each sample were ranked by their expression levels while excluding those with a median value of 0 in a training set. The median rank of each gene was then calculated in the two groups of the training set and top 200 genes were selected with the largest difference in the median rank between the LGG group and the GBM group (100 genes up-regulated in the LGG group and 100 genes up-regulated in the GBM group). The common DEGs obtained from the two training sets (TCGA dataset and GEO dataset) were selected as the candidate gene set to develop the gene-pair signature.

### Selection of Significantly Stable Gene Pairs and Reversely Stable Gene Pairs

The relationship between a pair of genes is denotated as *G*_1_ > *G*_2_ if the expression level of *G*_1_ is greater than that of *G*_2_ in a sample. In a cohort of *n* samples, the probability to observe this relative expression ordering (REO) (Li et al., 2016) in *m* samples can be represented using the binomial distribution model,

$$p=1-\overset{m-1}{\underset{i=0}{\sum }}\left(\begin{array}{c} n\\ i \end{array}\right)P_{0}^{i}{\left(1-P_{0}\right)}^{n-i}$$

where *P*_0_ (*P*_0_ = 50%) is the probability to observe the REO in a sample by chance. A gene pair with *p* < 0.01 was considered with a significantly stable REO.

A reversely stable gene pair refers to a gene pair which is significantly stable in both the GBM cohort and the LGG cohort but with the reversal REO directions, e.g., *G*_1_ > *G*_2_ in one cohort but *G*_1_ < *G*_2_ in the other.

### Consistency Assessment of Reversely Stable Gene Pairs

Candidate gene pairs with reversely stable REOs were identified in each train set and the overlapping pairs were considered as consistent reversal gene pairs. The binomial distribution model was used to evaluate the significance of consistency between the two groups of gene pairs.

### Classification Rules

Reclassification of a glioma sample into high-risk and low-risk groups is based on how many gene pairs in the signature showing the REO patterns of the GBM cohort using the majority voting rule. If more than 10 gene pairs among 21 pairs in a sample showing the REO patterns of GBM, the sample is classified as high-risk and also called as the GBM-like sample. Otherwise, it is reclassified as a low-risk sample.

### Survival Analysis

The Kaplan-Meier method was used to estimate the survival curves. The significance of the difference between two survival curves was tested using the log-rank method (Jones and Crowley, 1989). The Cox proportional-hazards model was used to evaluate the hazard ratios of the signature and other factors. All analyses were performed using Python 3.4.1 or R 3.5.1.

## Results

### Ambivalent Prognostic Impact of *TERT*-P Mutation

**FIGURE 1:**
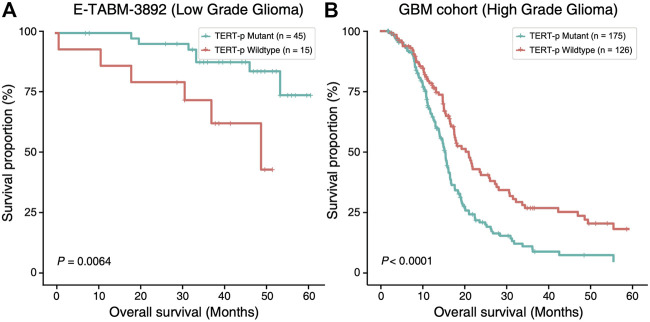
Ambivalent prognostic impact of *TERT*-p mutation. In LGGs (E-TABM-3892 dataset), **(A)** *TERT*-p mutant samples have a significantly longer OS than the wildtype samples whereas in GBMs (GBM cohort dataset), **(B)** *TERT*-p mutant samples have a significantly shorter OS.

In order to investigate the potential of *TERT* promoter mutation status as a prognostic factor, we compared the overall survival (OS) of *TERT*-p mutant and wildtype gliomas using E-MTAB-3892 (Kamoun et al., 2016) dataset and a GBM cohort from a previous study (Arita et al., 2016) (Figure 1). In LGGs, we observed a survival advantage of *TERT*-p mutant patients (*p* = 6.4E-03, Figure 1A). On the contrary, in the GBM cohort, *TERT*-p mutant patients showed a significantly shorter OS than the wildtype patients (*p* < 1E-04, Figure 1B). The median OS of low-grade samples with *TERT*-p mutations in the TCGA training dataset is shorter than 20 months, which leads us to suspect that there exist GBM-like patients in the low-grade *TERT*-p mutant samples.

### Development of the Classification Signature Consisting of 21 Gene-Pairs

**FIGURE 2:**
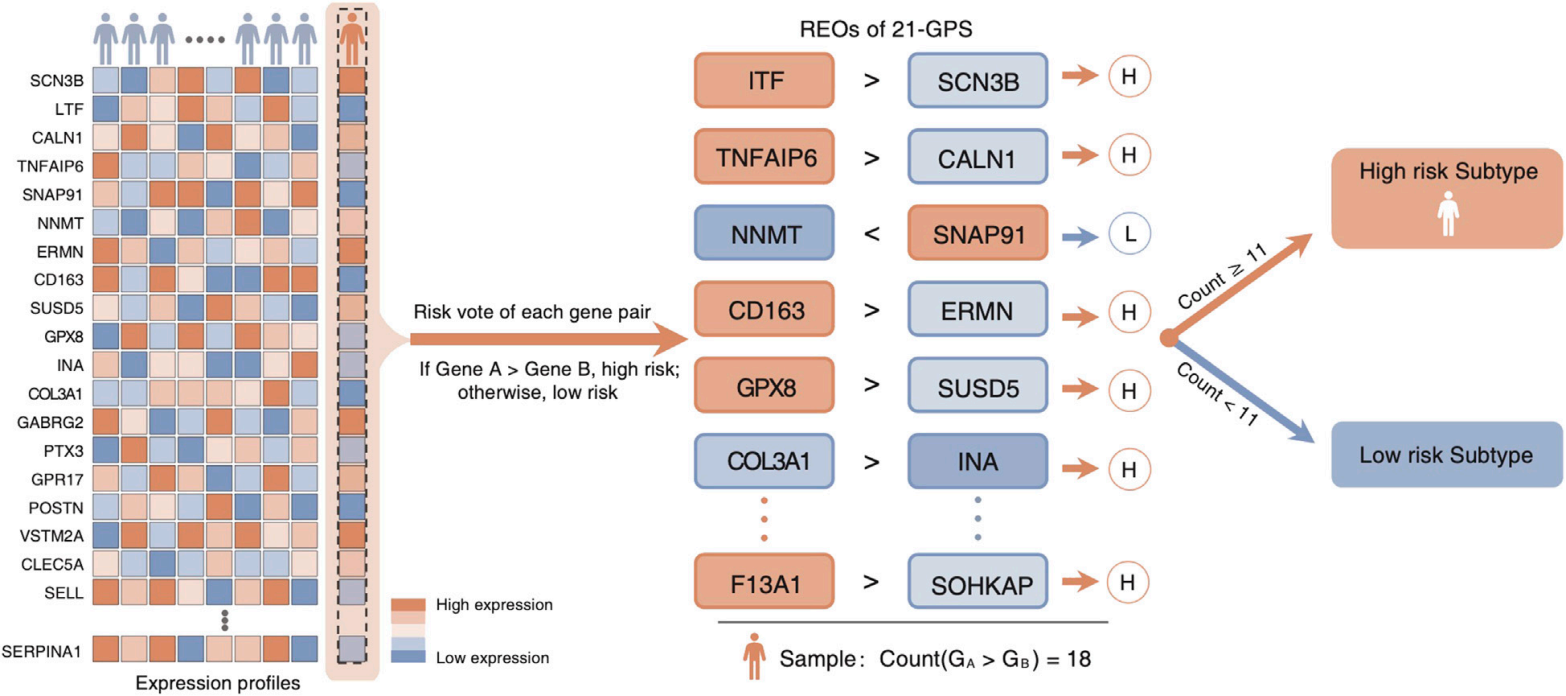
Classification scheme of the 21-GPS signature. For a given sample, we evaluate the REO of 21 gene pairs of the signature. If more than or equal to 11 pairs have a REO of gene A > gene B, which is the high-risk pattern, the sample is identified as a high-risk sample.

Top 200 DEGs with the largest differences in the median ranks between the LGGs and the GBMs were selected in the two training datasets, respectively. There exist 48 genes shared by the two training datasets with the same differential expression pattern. They were used to develop the classification signature based on REOs. From the GSE61374-GSE16011 dataset and the TCGA dataset, 559 and 485 reversal gene pairs were identified (adjusted *p* = 0.01), respectively, and with an intersection of 483 gene pairs. The maximum matching algorithm in graph theory is applied to remove the redundancy in the 483 pairs. Finally, 21 pairs of disjoint gene pairs were obtained, which are denoted as 21-GPS. For each gene pair in 21-GPS, the REO patterns (Gene A > Gene B) in Supplementary Table S2 are associated with a worse survival. If more than 10 gene pairs in a sample have these patterns, this sample was classified into the high-risk (GBM-like) group; otherwise, it was classified into the low-risk group. A scheme is given in Figure 2 to show the classification process.

### Significant Survival Difference Found Between the Two Groups Classified by 21-GPS

**FIGURE 3:**
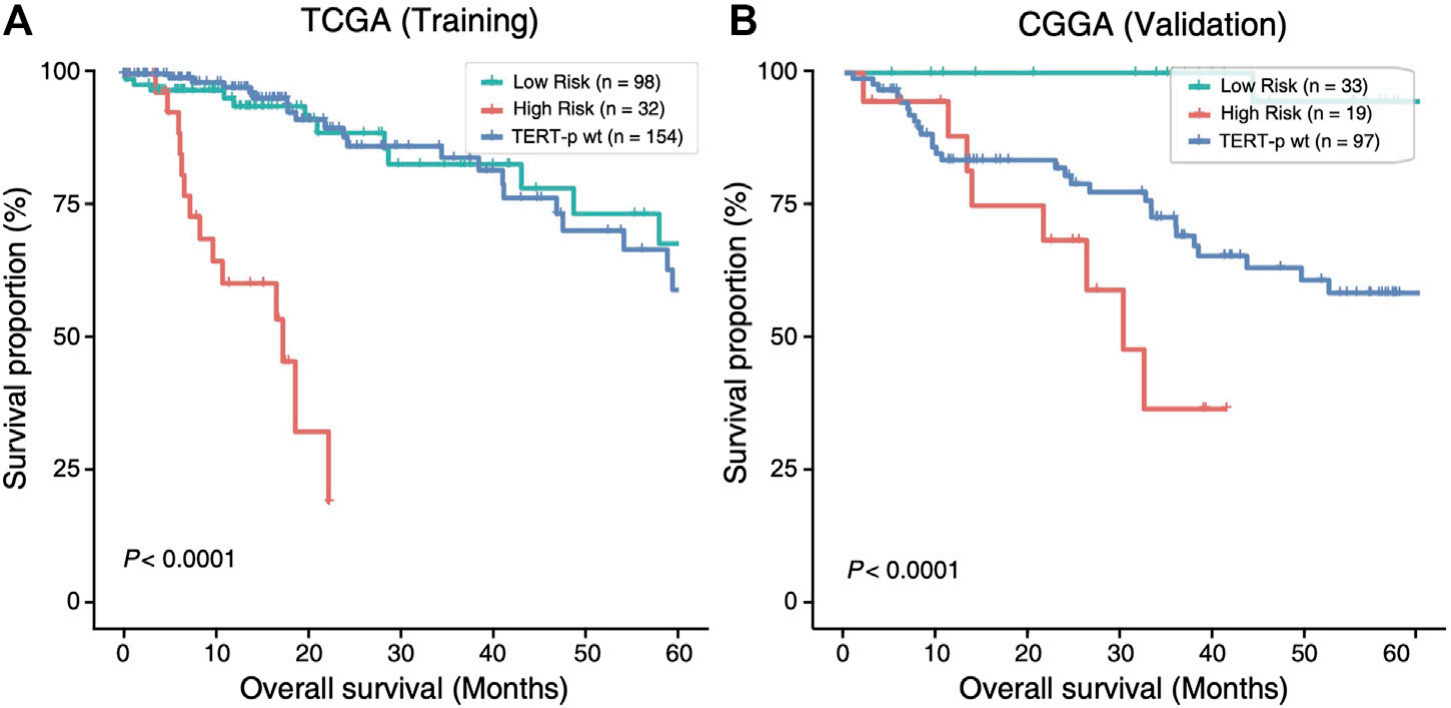
The high-risk samples identified by 21-GPS have significantly worse prognosis than the low-risk samples in the *TERT*-p mutant samples of the training TCGA dataset **(A)** and the validation CGGA dataset **(B)**. The survival curves of *TERT*-p wildtype samples are also shown for comparison.

The 21-GPS signature was first applied to the *TERT*-p mutant LGGs in both the TCGA training set and an independent validation dataset of CGGA (Figure 3). A group of high-risk samples was identified, respectively, in each dataset (32 in TCGA and 19 in CGGA), which even has a worse OS than the *TERT*-p wildtype samples. Furthermore, the OS is similar as that of the GBM samples. These samples should receive a more aggressive and early treatment.

### Survival Prediction Performance of 21-GPS for Samples Without *TERT*-P Status

**FIGURE 4:**
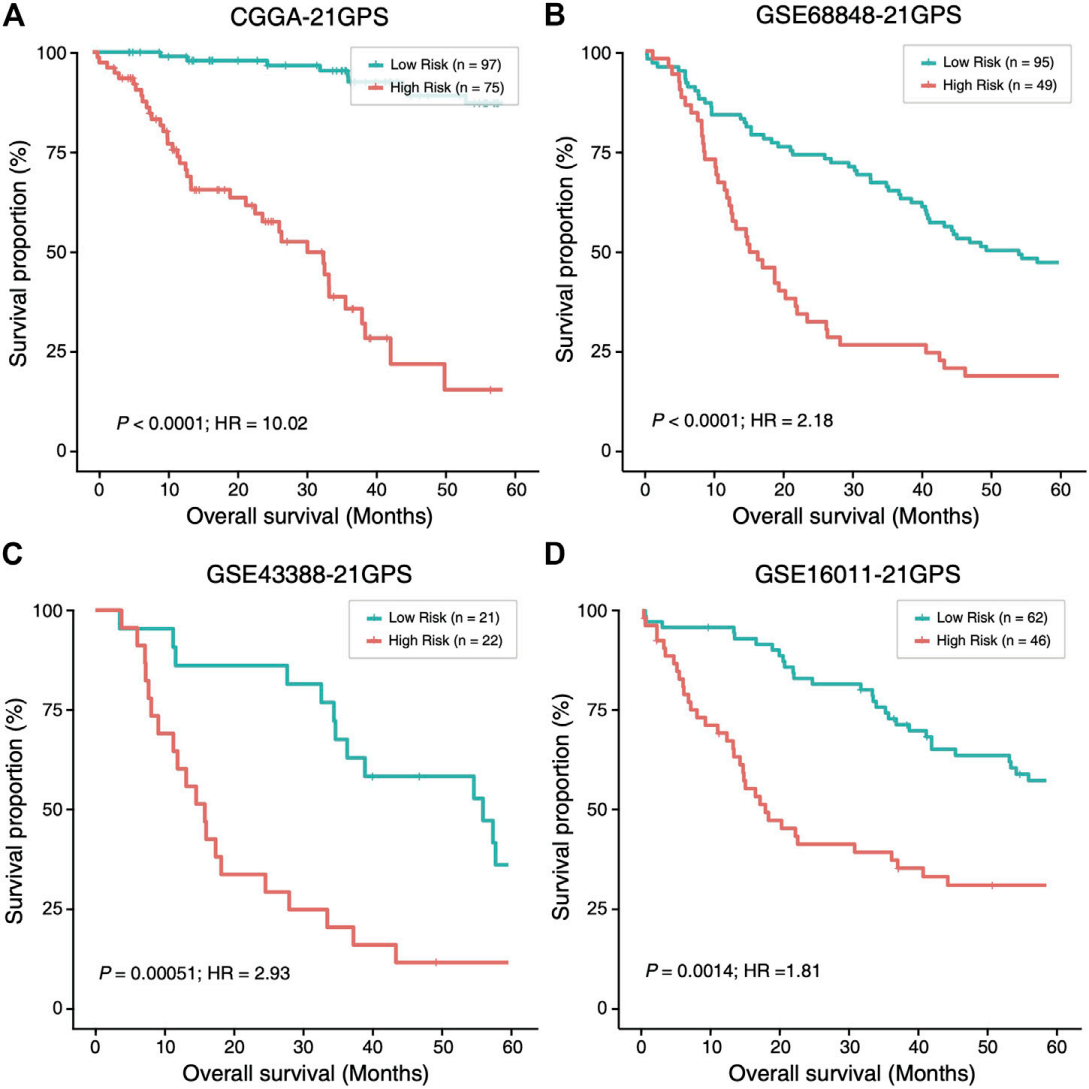
The high-risk samples identified by 21-GPS also have significantly worse prognosis than the low-risk samples in four independent datasets without the *TERT*-p mutation information, CGGA **(A)**, GSE68848 **(B)**, GSE43388 **(C)** and GSE16011 **(D)**. Hazard ratio (HR) associated with IDH mutation is comparing the wildtype ones with respect to the mutant ones in this figure and Figure 5.

In four datasets without *TERT*-p mutation status (Supplementary Table S1), 81, 53, 22 and 50 high-risk samples were identified, respectively, in CGGA, GSE68848, GSE43388 and GSE16011, using 21-GPS. All the high-risk samples have a significantly worse OS than the low-risk samples in each dataset (Figure 4). The median OS is shorter than 20 months in GSE68848, GSE43388 and GSE16011.

In the CGGA dataset, approximately 45% of LGGs were reclassified as high-risk samples. Among the high-risk samples, the proportion of *IDH* wildtype samples is significantly higher than that in the low-risk group (39.5 vs. 17.0%) and the proportion of grade III patients is also significantly higher (60.5 vs. 4.2%). Therefore, the high-risk group is enriched with grade III and *IDH* wildtype patients who have a greater survival risk.

### Higher Prognostic Value of 21-GPS Than *IDH* Mutation Status

**FIGURE 5:**
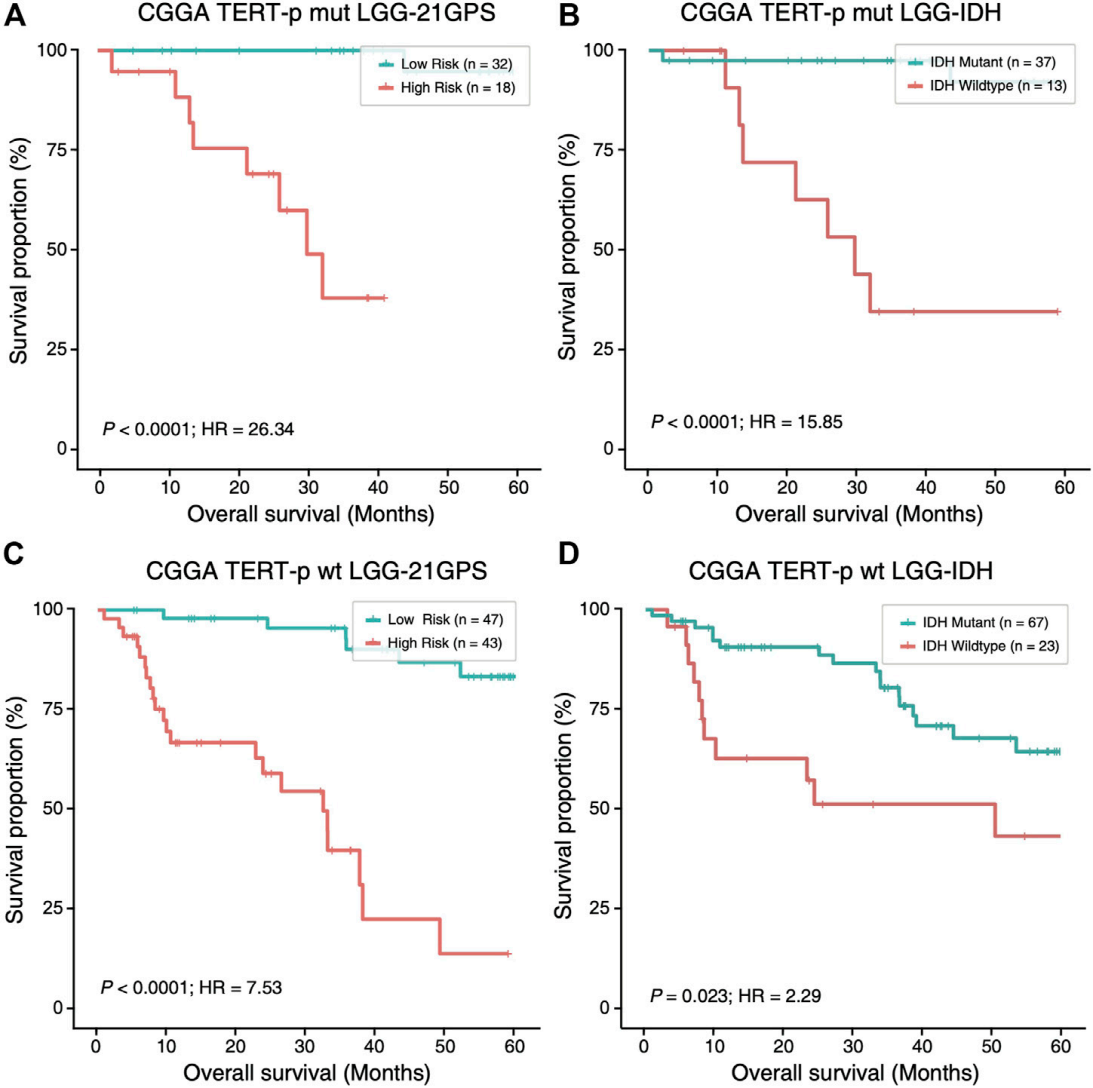
Comparison of the survival curves of the 21-GPS classification with the *IDH*-based classification in the *TERT*-p mutant samples **(A,B)** and wildtype samples **(C,D)**. The survival of the high-risk samples is significantly shorter than the low-risk samples in both the *TERT*-p mutated **(A)** and wildtype **(C)** LGGs. The survival of the *IDH* wildtype samples is also shorter than the *IDH* mutant samples in the *TERT*-p mutant LGGs or in the *TERT*-p wildtype LGGs **(D)**. But the hazard ratios and the significance levels are higher in the 21-GPS classification than the *IDH* classification.

Mutation statuses of *IDH* and *TERT* promoter have been used to classify gliomas in previous studies. Therefore, we sought to investigate the prognostic value of 21-GPS in comparison with the classification results based on the *IDH* and *TERT*-p mutation statuses using survival analysis. In the CGGA dataset of LGGs, a similar classification performance was observed between 21-GPS and *IDH* mutation status in the *TERT*-p mutant group (*p* < 0.0001 vs. *p* < 0.0001, log-rank test, Figures 5A,B). However, in the *TERT*-p wildtype samples, the 21-GPS classifier showed a much better classification performance than *IDH* mutation status (*p* < 0.0001 vs. *p* = 0.023, log-rank test, Figures 5C,D). In both the *TERT*-p mutant and wildtype groups, the 21-GPS classifier achieved higher hazard ratios than the *IDH* mutation status. Furthermore, we also carried out receiver operating characteristic (ROC) curve analysis to evaluate the classification performance of 21-GPS and *IDH* mutation status, using 3-year survival as the threshold to distinguish the reference high risk and low-risk groups (Supplementary Figure S1). The area under the ROC curve (AUC) is also higher for 21-GPS than for *IDH* mutation in both the *TERT*-p mutant group (0.955 vs. 0.886) and the *TERT*-p wildtype group (0.862 vs. 0.660). Thus, 21-GPS has a higher prognostic value than *IDH* mutation in stratifying LGGs.

### 21-GPS as an Independent Prognostic Factor Revealed by Cox Regression Analysis

Univariate and multivariate Cox regression models were used to evaluate the prognostic value of 21-GPS along common clinicopathological factors such as age, gender and *IDH* mutation status (Supplementary Table S3). In the TCGA and CGGA datasets, the Cox regression analysis were performed separately in the *TERT*-p mutant samples and wildtype samples. As the multivariate Cox regression result shows, the 21-GPS classification result is an independent prognostic factor in all five subsets, including both the training set and independent validation set (TCGA-LGG: HR = 11.33, *p* < 0.0001; CGGA: HR = 28.11, *p* < 0.0001; GSE16011-LGG: HR = 1.96, *p* = 0.0017; GSE68848-LGG: HR = 2.32, *p* < 0.0001; GSE43388-LGG: HR = 3.39, *p* = 0.0009). In the multivariate Cox regression result, the 21-GPS classification still shows an excellent prediction performance as an independent prognostic factor. The significance of 21-GPS in predicting OS is stronger than that of *IDH* mutation. In addition, the classification performance of 21-GPS is stable not only in the datasets obtained on RNA-sequencing platform and but also in the datasets obtained on microarray platforms.

### GBM-like Mutation Landscape Found in High-Risk Samples Identified by 21-GPS

**FIGURE 6:**
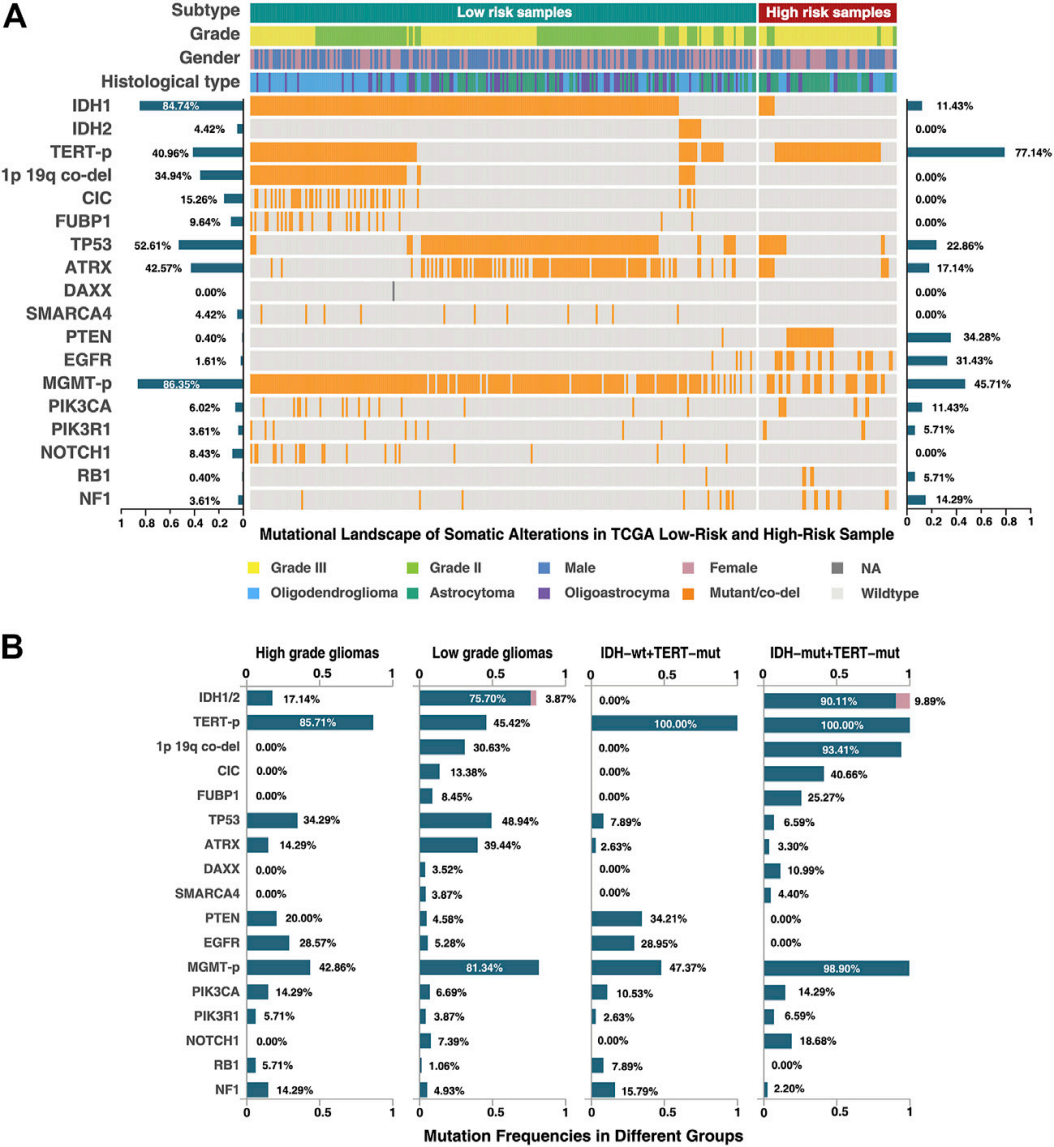
Mutational landscape of the low-risk and high-risk samples of the TCGA dataset classified by 21-GPS. The mutation frequencies are shown on the left-side for the low-risk group and on the right-side for the high-risk group in panel **(A)**. The mutation frequencies are also shown for the high-grade (GBM) and low-grade gliomas (left-two panels in **(B)**) and for the *IDH* wildtype and mutant groups in the *TERT*-p mutant panels (right two panels in **(B)**).

In order to further reveal the molecular characteristics of the high-risk group identified by 21-GPS, somatic mutational landscape is compared between the low-risk and high-risk samples in the TCGA dataset in which the somatic mutation information is available (Figure 6). In Figure 6, we selected the top 10 genes with mutation frequency greater than 3%, e.g., *IDH*, *TERT*-p and O-6-methylguanine-DNA methyltransferase promoter (*MGMT*-p) and some tumor-specific genes, e.g., tumor protein p53 (*TP53*) and phosphatase and tensin homolog (*PTEN*). The samples were grouped either by the original high- and low-grade labels (left two panels in Figure 6B) or by the combined *IDH* and *TERT*-p mutation statuses (right two panels in Figure 6B). For the latter classification method, only the *IDH* wildtype and *TERT*-p mutant subsets are displayed, which are expected to have the worst and best prognosis, respectively, among four possible subsets.

Among the low-risk samples, the proportion of *IDH1* and *IDH2* mutations, in a mutually exclusive state, is very high (91.6%). The ATRX chromatin remodeler gene (*ATRX*) and *TP53* show a tendency of co-mutation, similar as in low-grade astrocytoma. The proportions of malignant prognostic factors, e.g., mutations of *PTEN* and the epidermal growth factor receptor gene (*EGFR*), are very low. In short, the mutational landscape of the low-risk samples shows the classic molecular characteristics of LGGs.

In the high-risk group, we found that most of samples are *IDH* wildtype, and most of *IDH* mutant samples are accompanied with *ATRX* and *TP53* mutations or *TP53* mutations alone. The *TERT*-p mutation frequency is more than 80% in the high-risk group. *ATRX* mutations and *TERT*-p mutations show a mutually exclusive trend. In the WHO-2016 CNS classification system, primary GBM is characterized by *IDH* wildtype, and secondary GBM by *IDH* and *ATRX* mutations. If we only use the *IDH* and *TERT*-p mutation statuses to select the high-risk samples, a significant proportion of samples will be ignored, which can be identified by 21-GPS. Additionally, the landscape also revealed some interesting features in the high-risk samples. For example, *IDH* wildtype samples are frequently accompanied by *EGFR* mutations, and *PTEN* and *TP53* mutations tend to be mutually exclusive.

The mutation frequency plots of the high-risk group and the low-risk group (Figure 6A) are similar, respectively, with the high-grade and low-grade subsets. For example, in the high-grade or high-risk groups, the frequency of malignant mutations such as those of *PTEN*, *EGFR* and neurofibromin 1 (*NF1*), are higher while in the low-grade or low-risk groups the frequency of *IDH* mutations is higher. In the classification system based on the *IDH* and *TERT*-p mutation statuses, the samples with *ATRX* and *TP53* mutations are ignored, which represent a significant proportion of LGGs. *EGFR* mutations appear only in the *IDH* wildtype samples, but a proportion of *EGFR* mutated samples were found in the low-grade or low-risk groups. The prognostic value of these less-frequent mutations is ignored by the *IDH* and *TERT*-p mutation classification system, but not by our signature.

## Discussion and Conclusion

Diffuse glioma is a highly heterogeneous malignant tumor, which sees no much progress in its classification for more than 10 years (Filbin and Suva, 2016). With the advancement of high-throughput sequencing technologies, molecular markers have become important complements to histological features which make clinical classification of gliomas more accurate. However, we found that some low-grade gliomas have poor survival (less than 20 months), which may be related to inappropriate treatment. Previous study found that *TERT*-p mutation has a “bivalent” prognostic effect in diffuse gliomas (Arita et al., 2016). The low-grade gliomas especially oligodendrogliomas have the best prognosis and the mutation rate of *TERT*-p is close to 80%, while GBMs, which have the worst survival, also have a high mutation rate of more than 80% (Arita et al., 2013; Killela et al., 2014; Eckel-Passow et al., 2015). The mechanism of this opposite prognostic impact remains unexplained. One possible reason is due to high heterogeneity in gliomas and the classification based on the mutation status of a single-gene could lead wrong results. For example, if a tumor is mixed with *IDH*-mutated oligodendrogliomas and *IDH*-wildtype GBM, it will be classified as *IDH*-mutated oligodendrogliomas because the high sensitivity of next-generation sequencing technique enables us to detect the mutation at a very low tumor cell purity. But GBMs usually progress at a much higher rate and the tumor should be treated as GBM instead of oligodendroglioma. Our signature is based on the relative expression orderings of 21 gene pairs and uses the principle of majority voting to classify a sample. It has more robust performance than the classification based on genetic mutation statuses against tumor heterogeneity and sampling uncertainty.

Mutant (mut) or wildtype (wt) statuses of *IDH* and *TERT*-p have been used to classify gliomas into four categories (both mut, both wt, *IDH* mut and *TERT*-p wt, and *IDH* wt and *TERT*-p mut) (Zhang et al., 2015; Arita et al., 2016; Akyerli et al., 2018), which were shown significantly associated with prognosis. But this simple molecular classification does not consider the influence of tumor grades and cannot explain the biological mechanism of these two molecules. We show that the classification efficiency of *IDH* is not significant in the *TERT*-p wt samples. But our signature can classify both *TERT*-p wt samples and the mutation-status unknown samples robustly.

Using our signature to analyze the TCGA cohort, we found that the proportion of grade III samples is significantly greater than that of grade II in the high-risk group (Figure 6) and the *IDH* mutation frequency is significantly lower (15.9%) in the high-risk group than in the low-risk group while the frequencies of other malignant mutations are significantly higher. Among the high-risk samples, we found no samples with co-mutations of *IDH* and *TERT*-p, and those *IDH* mutated and *TERT*-p wildtype samples are usually accompanied by *ATRX* and *TP53* mutations. This is consistent with our understanding on GBM. In the *IDH* mutated high-risk samples, most of them are co-mutated with *ATRX*, which represents a typical molecular state of recurrent glioblastoma. In *IDH* wildtype high-risk samples, most of them carry the mutations of *PTEN* and/or *EGFR*, which are the characteristics of primary GBMs. The result is consistent with the telomere replacement extension mechanism discussed in previous studies (Heaphy et al., 2011; Akter and Kamijo, 2021; Rachakonda et al., 2021; Valenzuela et al., 2021). This indicates that our signature is capable of identifying the high-risk samples with either primary or recurrent malignant GBM characteristics.

In the fifth edition of the World Health Organization (WHO) Classification of Tumors of the Central Nervous System (WHO CNS5), which was released recently (Louis et al., 2021; Wen and Packer, 2021), *IDH* wildtype diffuse and astrocytic gliomas are classified as “GBM (*IDH* wildtype subtype)" with the presence of *TERT*-p mutation or *EGFR* gene amplification. This change reflects that there exist high-risk samples similar to GBM in grade II/III gliomas, even if they do not show the histological characteristics of GBM. In Figure 6, 77% of the reclassified high-risk group have *TERT*-p mutations, and 31.43% have *EGFR* mutations (*EGFR* mutation rate is only 1.61% in the low-risk group). This result is consistent with the new classification of WHO CNS5. In addition, in the low-risk group, almost all samples with *TERT*-p mutations are oligodendrogliomas, which characterizes the “oligodendroglioma, *IDH* mutant, *TERT*-p mutant” group in CNS5. In the reclassified TCGA cohort, approximately 90% of the high-risk group are *IDH* wildtype, and most of them were labeled as astrocytoma. In the other 10% of samples with *IDH* mutation did not include oligodendrogliomas, and they are likely to be classified as “Astrocytoma, *IDH*-mutant, grade IV” in CNS5. Therefore, our signature can screen out high-risk samples with GBM-like characteristics from all glioma samples, which has high potential in clinical application.

In conclusion, we developed a molecular signature based on the relative expression orderings of 21 gene pairs which can identify high-risk samples from gliomas, without relying on WHO grade and *IDH* status. These high-risk tumors show GBM-like characteristics and should receive more aggressive treatment. The 21-GPS has great potential to be applied to clinical diagnosis in the future.

## Data Availability

Publicly available datasets were analyzed in this study. This data can be found here: The datasets GSE16011, GSE61374, GSE68848, GSE43388 for this study can be found in the Gene Expression Omnibus, https://www.ncbi.nlm.nih.gov/geo/. The datasets TCGA for this study can be found in The Cancer Genome Atlas, https://cancergenome.nih.gov/. The datasets CGGA for this study can be found in the Chinese Glioma Genome Atlas, http://www.cgga.org.cn/. The datasets E-MTAB-3892 for this study can be found in ArrayExpress, https://www.ebi.ac.uk/arrayexpress/.
