# Supplementary material for: A Qualitative Signature to Identify TERT Promoter Mutant High-Risk Tumors in Low-Grade Gliomas
Source: Front Mol Biosci. 2022 Apr 14;9:806727. doi: 10.3389/fmolb.2022.806727 (PMC9047542; doi:10.3389/fmolb.2022.806727)
Supplement: Supplementary file 1 [file DataSheet1.docx]

Supplementary Materials

# Supplementary Figures and Tables

**
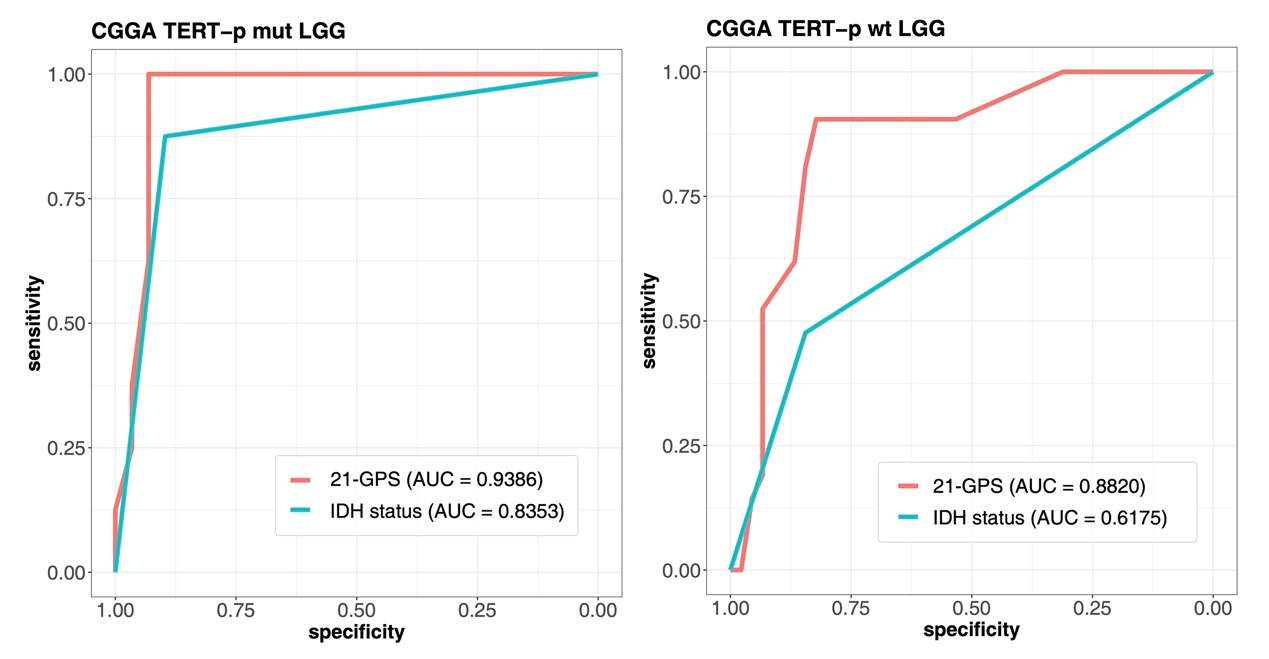
**

**Figure S1.** Receiver operating characteristic (ROC) curves based on the 21-GPS classification and the *IDH* mutation status classification in the *TERT*-p mutant group (left) and wildtype group (right). The samples with an OS longer than 3 years were considered as the reference low-risk group while the samples losing follow-up within 3 years were excluded from this analysis. In both the subsets, the 21-GPS shows a better performance than *IDH*.

**Table S1.** Clinical characteristics of the validation datasets

|  | **CGGA**  **(*n* = 310)** | **GSE16011 LGG**  **(*n* = 106)** | **GSE68848**  **(*n* = 183)** | **GSE43388**  **(*n* = 43)** |
| --- | --- | --- | --- | --- |
| Grade and Histone Type — no.(%) | | | | |
| Grade II |  |  |  |  |
| Oligodendroglioma | 27 (25.7) | 8 (33.3) | 30 (30.3) | 0 (0.0) |
| Oligoastrocytoma | 35 (33.3) | 3 (12.5) | 4 (4.0) | 0 (0.0) |
| Astrocytoma | 43 (41.0) | 13 (54.2) | 65 (65.7) | 0 (0.0) |
| Grade III |  |  |  |  |
| Oligodendroglioma | 12 (17.9) | 41 (50.0) | 23 (27.4) | 26 (60.5) |
| Oligoastrocytoma | 32 (47.8) | 25 (30.5) | 3 (3.6) | 17 (39.5) |
| Astrocytoma | 23 (34.3) | 16 (19.5) | 58 (69.0) | 0 (0.0) |
| Grade IV |  |  |  |  |
| Glioblastoma | 138 (-) | 0 (-) | 0 (-) | 0 (-) |
| Age at diagnosis — years | | | | |
| Mean | 43.4±12.1 | 46.5±14.0 | 43.8±15.1 | 45.5±12.8 |
| Range | 8-81 | 24-82 | 15-75 | 18-66 |
| Male sex — no./total (%) | 195/310 (53.0) | 71/105 (58.9) | - | 2743 (63.6） |
| *IDH* status — no. (%) | | | | |
| *IDH1/2*-mut | 151 (62.4) | 46(98.0) | - | 12 (87.5) |
| *IDH*-wt | 134 (36.9) | 40 (12.0) | - | 15 (12.5) |
| 1p19q status — no. (%) | | | | |
| co-del | - | 36 (1.3) | - | - |
| no co-del | - | 34 (98.7) | - | - |
| Median survival — months | 16.3 | 38.9 | 23.7 | 49.0 |

**Table S2.** Gene pairs of the 21-GPS signature

| **GENE A** | **GENE ID** | **GENE B** | **GENE ID** |
| --- | --- | --- | --- |
| *LTF* | 4057 | *SCN3B* | 55800 |
| *TNFAIP6* | 7130 | *CALN1* | 83698 |
| *NNMT* | 4837 | *SNAP91* | 9892 |
| *CD163* | 9332 | *ERMN* | 57471 |
| *GPX8* | 493869 | *SUSD5* | 26032 |
| *COL3A1* | 1281 | *INA* | 9118 |
| *PTX3* | 5806 | *GABRG2* | 2566 |
| *POSTN* | 10631 | *GPR17* | 2840 |
| *CLEC5A* | 23601 | *VSTM2A* | 222008 |
| *SERPINA1* | 5265 | *SELL* | 6402 |
| *IL13RA2* | 3598 | *SYT4* | 6860 |
| *CCNB2* | 9133 | *CACNG2* | 10369 |
| *ANXA1* | 301 | *NOG* | 9241 |
| *MOXD1* | 26002 | *LUZP2* | 338645 |
| *RBP1* | 5947 | *CA10* | 56934 |
| *SRPX2* | 27286 | *ZNF488* | 118738 |
| *TCIM* | 56892 | *CRTAC1* | 55118 |
| *HOXA5* | 3202 | *GABRB3* | 2562 |
| *SERPINE1* | 5054 | *CARNS1* | 57571 |
| *PDPN* | 10630 | *F5* | 2153 |
| *F13A1* | 2162 | *SPHKAP* | 80309 |

**Table S3**. Univariate and multivariate Cox regression analysis results in different datasets. Grade II, female, *IDH* wt, low-risk samples were used, respectively, as the reference for grade, gender, *IDH* mutation status and 21-GPS factors.

| Dataset | Univariate (OS) | | | | | | | Multivariate (OS) | | | | | |
| --- | --- | --- | --- | --- | --- | --- | --- | --- | --- | --- | --- | --- | --- |
|  |  | *TERT*-p mut | | | *TERT*-p wt | | | *TERT*-p mut | | | *TERT*-p wt | | |
|  |  | HR | 95%C.I. | *p*-value | HR | 95%C.I. | *p*-value | HR | 95%C.I. | *p*-value | HR | 95%C.I. | *p*-value |
| TCGA-LGG | Grade | 5.35 | 2.71-10.55 | 1.28E-06 | 3.17 | 1.59-6.33 | 0.001 | 2.23 | 0.92-5.42 | 0.0771 | 2.63 | 1.24-5.56 | 0.01 |
|  | Age | 1.10 | 1.06-1.13 | 9.22E-09 | 1.04 | 1.01-1.07 | 0.0035 | 1.08 | 1.04-1.12 | 3.76E-05 | 1.06 | 1.03-1.08 | 8.43E-05 |
|  | Gender | 1.03 | 0.49-2.15 | 0.94 | 1.04 | 0.51-2.11 | 0.92 | 1.07 | 0.54-2.10 | 0.8462 | 0.86 | 0.40-1.85 | 0.70 |
|  | *IDH* | 0.06 | 0.02-0.14 | 2.55E-10 | 0.24 | 0.11-0.55 | 0.00066 | 0.38 | 0.08-1.76 | 0.2144 | 0.30 | 0.12- 0.76 | 0.01 |
|  | 21-GPS | 11.33 | 5.34-24.05 | 2.55E-10 | 4.64 | 1.86-11.59 | 0.001 | 3.45 | 0.77-15.47 | 0.105 | 4.19 | 1.60-10.94 | 0.0035 |
| CGGA | Grade | 26.33 | 3.59-192.90 | 0.0012 | 4.27 | 2.20-8.28 | 1.72E-05 | 4.98 | 0.53-46.94 | 0.1604 | 1.32 | 0.60-2.90 | 0.48 |
|  | Age | 1.03 | 1.00-1.07 | 0.048 | 1.04 | 1.01-1.07 | 0.003 | 1.01 | 0.98-1.05 | 0.5346 | 1.02 | 0.99-1.05 | 0.23 |
|  | Gender | 1.05 | 0.53-2.04 | 0.89 | 1.07 | 0.59-1.91 | 0.85 | 0.76 | 0.37-1.54 | 0.4446 | 0.77 | 0.42-1.43 | 0.42 |
|  | *IDH* | 0.38 | -0.07-0.31 | 5.04E-07 | 0.27 | 0.15-0.49 | 1.71E-05 | 0.50 | 0.21-1.18 | 0.1152 * | 0.63 | 0.31-1.28 | 0.19 |
|  | 21-GPS | 28.11 | 8.06-98.08 | 1.67E-07 | 14.53 | 6.10-34.61 | 1.48E-09 | 10.10 | 2.27-44.93 | 0.0024** | 10.57 | 3.90-28.65 | 3.55E-06 |
|  | All LGG Samples (no TERT promoter status) | | | | | | | All LGG Samples (no TERT promoter status) | | | | | |
|  |  | HR | 95%C.I. | *p*-value |  | | | HR | 95%C.I. | *p*-value |  | | |
| GSE16011-LGG | Grade | 1.19 | 0.68-2.08 | 0.55 |  | | | 0.77 | 0.40-1.45 | 0.41 |  | | |
|  | Age | 1.04 | 1.02-1.05 | 8.58E-05 |  | | | 1.04 | 1.02-1.06 | 1.93E-05 |  |  |  |
|  | Gender | 0.95 | 0.61-1.47 | 0.80 |  | | | 1.08 | 0.64-1.80 | 0.79 |  |  |  |
|  | *IDH* | 0.84 | 0.51-1.33 | 0.43 |  | | | 1.06 | 0.61-1.83 | 0.85 |  |  |  |
|  | 21-GPS | 1.96 | 1.29-2.99 | 0.0017 |  | | | 2.44 | 1.37-4.37 | 0.0026 |  |  |  |
| GSE68848-LGG | Grade | 1.29 | 0.93-1.79 | 0.14 |  | | | 1.02 | 0.71-1.47 | 0.92 |  | | |
|  | Age | 1.04 | 1.03-1.06 | 3.98e-09 |  | | | 1.04 | 1.03-1.06 | 3.55e-08 |  |  |  |
|  | Gender | - | - | - |  | | | - | - | - |  |  |  |
|  | *IDH* | - | - | - |  | | | - | - | - |  |  |  |
|  | 21-GPS | 2.33 | 1.62-3.34 | 4.51e-06 |  | | | 2.43 | 1.62-3.65 | 1.80e-05 |  |  |  |
| GSE43388-LGG | Grade | - | - | - |  | | | - | - | - |  | | |
|  | Age | 1.02 | 0.98-1.04 | 0.39 |  | | | 1.02 | 0.98-1.06 | 0.38 |  |  |  |
|  | Gender | 0.94 | 0.48-1.87 | 0.87 |  | | | 1.75 | 0.64-4.76 | 0.27 |  |  |  |
|  | *IDH* | 0.10 | 0.03-0.33 | 0.00011 |  | | | 0.04 | 0.01-0.21 | 0.00016 |  |  |  |
|  | 21-GPS | 3.40 | 1.65-6.99 | 0.0009 |  | | | 0.34 | 0.09-1.30 | 0.12 |  |  |  |
